# Detecting Protein Complexes in Protein Interaction Networks Modeled as Gene Expression Biclusters

**DOI:** 10.1371/journal.pone.0144163

**Published:** 2015-12-07

**Authors:** Eileen Marie Hanna, Nazar Zaki, Amr Amin

**Affiliations:** 1 Intelligent Systems, College of Info. Tech., UAEU, Al Ain 17551, UAE; 2 Department of Biology, College of Science, UAEU, Al Ain 15551, UAE; 3 Faculty of Science, Cairo University, Cairo, Egypt; Koç University, TURKEY

## Abstract

Developing suitable methods for the detection of protein complexes in protein interaction networks continues to be an intriguing area of research. The importance of this objective originates from the fact that protein complexes are key players in most cellular processes. The more complexes we identify, the better we can understand normal as well as abnormal molecular events. Up till now, various computational methods were designed for this purpose. However, despite their notable performance, questions arise regarding potential ways to improve them, in addition to ameliorative guidelines to introduce novel approaches. A close interpretation leads to the assent that the way in which protein interaction networks are initially viewed should be adjusted. These networks are dynamic in reality and it is necessary to consider this fact to enhance the detection of protein complexes. In this paper, we present “DyCluster”, a framework to model the dynamic aspect of protein interaction networks by incorporating gene expression data, through biclustering techniques, prior to applying complex-detection algorithms. The experimental results show that DyCluster leads to higher numbers of correctly-detected complexes with better evaluation scores. The high accuracy achieved by DyCluster in detecting protein complexes is a valid argument in favor of the proposed method. DyCluster is also able to detect biologically meaningful protein groups. The code and datasets used in the study are downloadable from https://github.com/emhanna/DyCluster.

## Introduction

Protein complexes are groups of interacting proteins associated to specific cellular functions [1] and they are fundamental players in almost all biological processes. The identification of the complexes incorporated in a protein-protein interaction (PPI) dataset is indeed highly beneficial. One of the ultimate goals of this scenario is to be able to associate protein complexes with normal molecular events, and subsequently, to link the occurrence of inconsistent processes with different diseases. Undoubtedly, such knowledge could lead to the development of more effective therapies. The experimental methods designed to study the PPI and incorporated complexes, such as yeast two-hybrid (Y2H) [2] and tandem affinity purification (TAP-MS) [3] approaches, are vulnerable to high error rates [4] and practically limited in terms of time and cost. As a result, various computational methods were developed to complement and to reduce the required experimental efforts. A graph *G* = (*V*, *E*) is conventionally used to represent proteins *V* and their interconnections *E* as nodes and edges, respectively. Such representation was, and still is, the basis of many computational methods seeking to accurately describe and to identify enclosed protein-complex structures. A large number of these methods are based on the assumption that protein complexes correspond to dense and highly-interconnected sub-graphs. Among those methods, we here point out: Markov Clustering (MCL) [5] which uses random walks in protein interaction networks; the molecular complex detection (MCODE) algorithm [6] which considers complexes as dense regions grown from highly-weighted vertices; the clustering based on maximal cliques (CMC) method [7]; the Affinity Propagation (AP) algorithm [8]; ClusterONE [9] which identifies protein complexes by clustering with overlapping neighborhood expansion; the restricted neighborhood search (RNSC) algorithm [10, 11]; and CFinder [12] which is based on the clique percolation method. Other approaches which are not centered on the density notion were also presented; namely: ProRank [13, 14] and ProRank+ [15] which mainly use a protein ranking algorithm to identify essential proteins in a PPI network and form complexes accordingly; and finally PEWCC [16] which assesses the reliability of PPI data based on the weighted clustering coefficient notion prior to detecting protein complexes.

When compared to reference sets of biologically-identified protein complexes, most of the introduced computational approaches could achieve good complex-detection rates with adequate evaluation scores. Certainly, the higher their accuracy levels, the more they are reliable and the more likely they can be utilized by scientists and biologists. The improvements of protein-complex detection algorithms as well as the design of novel approaches seem to meet at the notion of reforming the way in which a PPI dataset is initially represented. PPI networks are in fact dynamic [17]. Hence, the shift from viewing PPI networks as static to modeling the dynamicity of these networks became fundamental [18]. This adaptation can currently be acquired thanks to the amounts and the diversity of biological information, whether temporal, spatial or contextual, generated by advanced experimental techniques such as ChIP-chip [19] and ChIP-seq [20].

In this paper, first we emphasize the advantages of shifting to dynamic PPI networks, specifically when it comes to the problem of detecting protein complexes; then we underline possible approaches to model the dynamic aspect of protein interactions and we highlight some of the existing methods. Second, we introduce “DyCluster”, a framework for the detection of protein complexes in dynamic PPI networks modeled using gene expression data, through biclustering techniques. Finally, we present our experimental study which shows that the results generated by applying complex-detection methods based on our framework are better than those corresponding to the methods applied on static PPI networks, in terms of the number of matched complexes, accuracy and other evaluation scores. An additional experiment on biological data is also presented.

## Dynamic PPI Networks

As an inter-disciplinary research area, computational biology is expected to profit from the continuous growth and diversity of biological data collected using advanced experimental techniques. Such information includes, but is not limited to, gene expression data [21] which report quantitative measurement of RNA species in cellular compartments across various conditions; sub-cellular localization annotations [22] which provide spatial positions of elements in cellular components; and gene ontology annotations [23] which highlight genes that are present across different species. The enrichment of biological representations, and particularly PPI networks, using such data types indeed allows better replication of real cellular events through the modeling of temporal, spatial and contextual dynamics which describe and influence cellular processes [24–26]. When the dynamics controlling the occurrence of protein interactions are included in PPI networks, the analytical results, and namely the detected protein complexes in such network variants, would potentially be more accurate. Since PPI datasets are generated by experimental techniques that are liable to high error rates [4], the computational methods designed to explore them are also susceptible to those errors. Various filtering techniques were thus proposed to pre-process PPI data before analyzing them, such as FSWeight [27], AdjustCD [28] and PE-measure [16]. Nevertheless, issues also exist in other biological information, such as gene expression data, which have yet low protein coverage in contrast with PPI datasets that are typically very large. Despite that, the combination of different descriptive biological data may be considered as a search for evidence intersection. The higher the recurrence of information and/or inferences in experimental results, the better could be our confidence that they exist in reality. Consequently, dynamic PPI networks, modeled using various experimental data, could verify or possibly contradict known biological concepts and may as well uncover previously-unknown biological facts. Different kinds of information could be drawn when exploring a PPI data set. Nonetheless, the categorization of such data is generally not simple; as in the case of distinguishing between protein complexes and functional modules, for example. In fact, complexes are formed by proteins which interconnect at the same time and place, whereas the members of functional modules may interact at different times and places [29]. Accordingly, by incorporating spatiotemporal information drawn from gene expression and sub-cellular localization annotations datasets, for instance, such classification of network modules can be acquired. Similarly, the biological enrichment of a PPI network potentially allows the identification of protein sub-complexes. Many methods were developed to solve this important research problem, but they all apply to static PPI networks [30]. The inclusion of temporal, spatial and contextual attributes, which guide PPIs, can lower the rates of false positives and false negatives at the level of the detected complexes and their protein members as well. In other words, these attributes can be used to cluster the proteins and their interconnections based on the conditions which govern them. A protein complex-detection method shall be applied on the clusters, with a generalization capability indeed. Consequently, the overall accuracy of the produced results would be better. The former potentially applies to other exploratory approaches of PPI networks.

**Fig 1:**
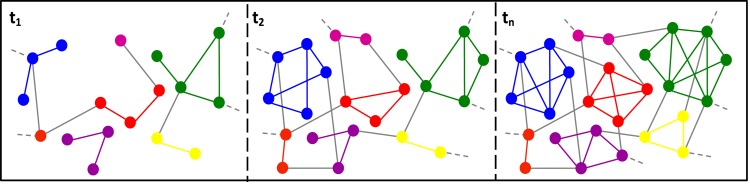
Snapshots of a hypothetical PPI network, showing its dynamics through different temporal, spatial and/or contextual settings. Nodes and edges of the same color belong to the same protein complex.

Instead of a single and comprehensive representation of a PPI dataset, by incorporating conditionality features of PPI events, we would rather be looking at a series of snapshots of a PPI network modeled based on either one or a combination of temporal, spatial and contextual settings (Fig 1). The interpretation of a dynamic interaction network and its state transitions depends on the types of data which are used to biologically-condition PPI events.

Gene expression data report quantities of RNA across different time points in cellular processes. It is believed that genes with correlated expressions across different conditions most likely interact. The combination of gene expression information with PPI data to model the dynamics of the corresponding PPI networks could potentially reveal the processes which underline the formation of protein complexes. For instance, Wang et al. [31] showed that a just-in-time mechanism elapsing through continuous time points delineates the formation of most complexes. The statistical 3-sigma principle was then used by the works presented in [31] and [32] to define the active time points of proteins based on their gene expression levels and consequently, introduce approaches to detect and refine protein complexes. The core-attachment interpretation of complexes was recently adopted in [33]; based on the dynamics inferred by gene expression data, the identification of a protein complex is split into two main parts: a static core consisting of proteins expressed throughout the whole cell cycle and a short-lived dynamic attachment. The results achieved by these approaches were better than the ones based on static PPI networks. Kim et al. [24] highlighted some of the computational methods used to infer dynamic networks from expression data based on statistical dependence to classify nodes and edges as active or inactive. These methods include: Bayesian networks [34], relevance networks [35], Markov Random Fields [36], ordinary differential equations [37] and logic-based models [38]. Since it is favorable to incorporate the spatial dynamics towards improving complex-detection approaches, various methods were designed to study the spatial movements of proteins [25]. However, in addition to mathematical modeling techniques, further approaches to appropriately integrate spatial protein dynamics in PPI networks are still required. By providing information about genes that are shared across species, gene ontology annotations can also be used to model the dynamics of PPI networks [26]. As an indicator of interaction probability, various weighting schemes were introduced to assign PPI weights based on the similarity degrees of gene ontology terms between interacting partners. Among these approaches are SWEMODE [39], which detects communities within PPI networks based on weighted clustering coefficient and weighted average nearest-neighbors degree measures, and OIIP [26], which is a method to detect protein complexes in PPI networks by assigning node and edge weights based on the size of gene annotations.

Modeling the dynamics of PPI networks through the integration of biological attributes particularly enhances the computational methods designed to detect protein complexes. It not only participates in uncovering the mechanisms of protein-complex formation but also points out useful details for the design of such methods. In addition, the former may help categorize protein complexes and could be informative regarding their building blocks as well.

## Methods

We hereafter present DyCluster, a framework for detecting protein complexes in dynamic PPI networks modeled using gene expression data through biclustering techniques. Our framework requires a gene expression dataset and a PPI dataset. It consists of five main steps:

1. Biclustering the gene expression data
2. Extracting the biclusters’ PPIs from an assigned PPI dataset
3. Pruning the biclusters’ PPIs
4. Detecting the protein complexes
5. Merging and filtering the sets of detected protein complexes

**Fig 2:**
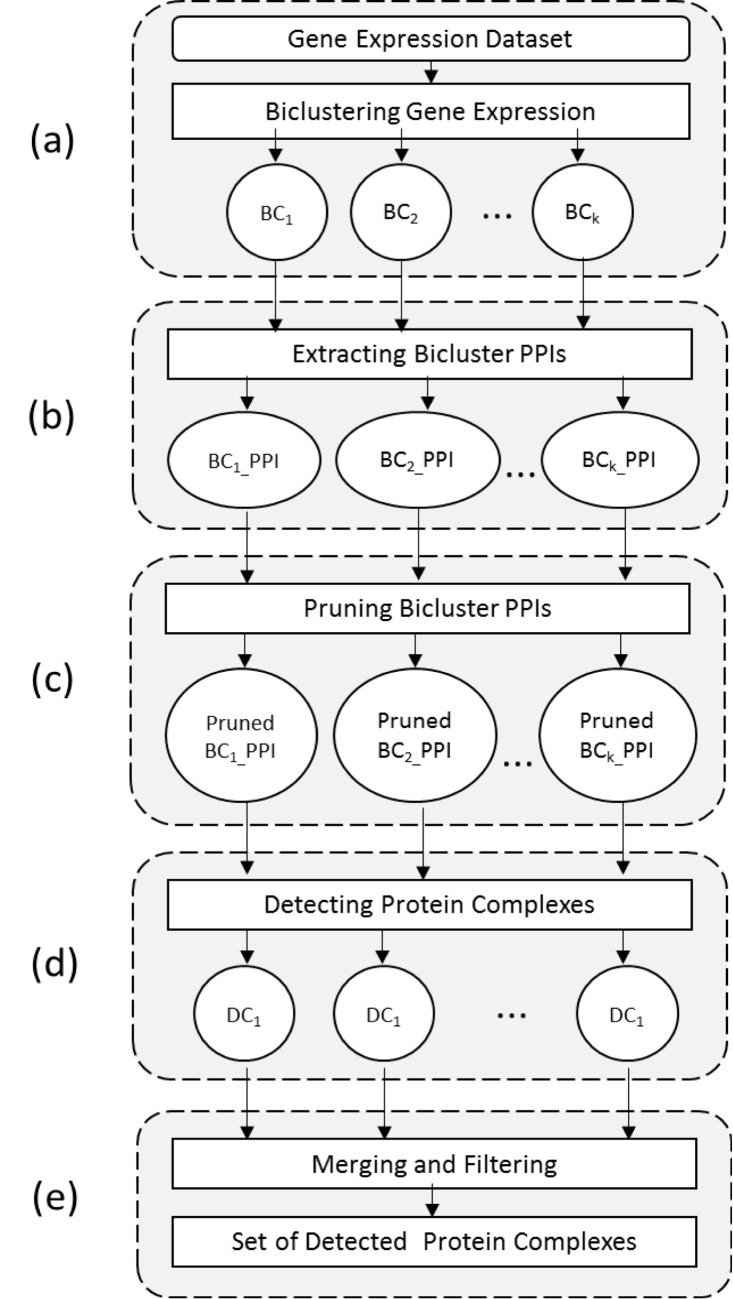
An outline of the DyCluster framework developed for the detection of protein complexes in dynamic PPI networks modeled as gene expression biclusters.

An outline of the approach is presented in Fig 2.

### Biclustering Gene Expression Data

A gene expression dataset reports the expression levels of a large number of genes across different environmental conditions, time points, organs, species, etc. It is conventionally represented as a matrix in which rows and columns correspond to genes and their expression levels at different conditions (samples), respectively. Various methods were developed to analyze gene expression data under the assumption that the ones which exhibit similar expression patterns across a set of conditions are more likely functionally-related [40]. The analysis of these datasets is challenging because they are usually unbalanced, i.e. the number of genes is quite larger than the number of conditions [41]. Many approaches were proposed to group genes according to their expression patterns; in particular, data mining approaches such as classification and clustering. Classification methods require knowing the label of the resulting classes in advance. Several research efforts were invested in studying the application of such supervised techniques on gene expression data [42]. However, the prior suggestion of classes somehow limits the process of data exploration. On the other hand, typical clustering techniques have two drawbacks when applied on gene expression data [43]: first, each gene must be grouped into a cluster even if its similarity with the cluster members is relatively low; and second, a gene can belong to one cluster only. Consequently, classical clustering methods cannot fully handle gene expression data since they do not account for the fact that a large number of genes can exhibit multiple biological functions [44], and thus can belong to more than one cluster. Besides, clustering spans the whole samples set whereas in reality, the expression patterns of a gene cluster may be correlated based on a subset of samples only. It is actually expected to produce groupings of co-expressed elements under subsets of conditions whose expression patterns are presumably independent across the rest of the conditions.

Thanks to the simultaneous two-dimensional clustering capability which they provide, biclustering techniques presented better means to explore expression data [45, 46]. Actually, they allow the identification of subsets of co-regulated genes across subsets of samples. And in analogy to biological facts, a gene may belong to multiple clusters and a gene may not fit in any cluster in some cases. A formal problem formulation of biclustering gene expression data is as follows: Let *A* be an n*m data matrix representing a gene expression dataset consisting of *n* genes measured across *m* conditions, *a*_*ij*_ is a real value corresponding to the expression level of the gene at row *i* and the condition at column *j*. The goal is to find a set of biclusters *BC*(*I*, *J*); where *I* is a subsets of genes which exhibit similar expression patters across the subset of conditions *J*.

We hereafter, highlight some of the existing biclustering approaches which will also be used at later stages to evaluate DyCluster. The first application of biclustering on gene expression data was conducted by Cheng and Church [47]. They presented a method (CC) consisting of a greedy search heuristic to form the biclusters, namely the set covering algorithm, and relying on the Mean Square Residue (MSR) measure to assess their quality based on a specified threshold. The MSR of a bicluster BC, of *I* rows and *J* columns, reflects the degree of coherence of the genes and the conditions that it includes (as shown in Eq (1)).

$$\begin{array}{c} MSR\left(BC\right)=\frac{1}{\left|I||J\right|}\sum_{i=1}^{\left|I\right|}\sum_{j=1}^{\left|J\right|}{\left(bc_{ij}-bc_{iJ}-bc_{Ij}+bc_{IJ}\right)}^{2} \end{array}$$ (1)

where *bc*_*ij*_, *bc*_*iJ*_, *bc*_*Ij*_ and *bc*_*IJ*_ represent the elements in row *i* and column *j*, the row and the column means, and the mean of *BC*, respectively. The lower the MSR, the higher is the bicluster coherence. Correlations among genes can be expressed in terms of scaling and shifting patterns. One aspect of the robustness of a biclustering algorithm, when applied on expression data, is in its ability to capture both types of patterns. MSR can only detect shifting correspondences among the expression levels of genes [48]. Despite that, it has been adopted by several similar approaches and some variants of this measure were also introduced to identify scaling patterns [49]. Other methods, that do not use metrics to evaluate the formed groupings throughout their operations, were also developed. The Order Preserving Sub Matrix (OPSM) algorithm [50] searches for large sub-matrices in which genes have the same linear ordering of the samples. The Iterative Signature Algorithm (ISA) [51] uses the signature algorithm to identify self-consistent transcriptional modules consisting of co-expressed genes and the samples corresponding to them. A comprehensive survey of these methods and others can be found in [45].

Given a gene expression dataset, the first stage of our framework involves biclustering these data into subsets of genes which exhibit similar variations in their expression levels across subsets of conditions, as shown in Fig 2(a).

### Extracting Biclusters’ PPI Data

Given the generated set of gene biclusters as shown in Eq (2):

$$\begin{array}{c} BC=\{BC_{1}\left(I_{1},J_{1}\right),BC_{2}\left(I_{2},J_{2}\right),...,BC_{k}\left(I_{k},J_{k}\right)\} \end{array}$$ (2)

The next step consists of finding the interconnections among the members of each bicluster based on a specified PPI dataset. The interactions in the PPI dataset which involve elements belonging to the set of proteins, *P*_*l*_ = {*p*_*l*1_, *p*_*l*2_, …, *p*_*lI*_*l*__}, contained in *BC*_*l*_(*I*_*l*_, *J*_*l*_), are added to the sub-PPI dataset, *BC*_*l*_(*I*_*l*_, *J*_*l*_)_*PPI*, corresponding to this bicluster. The sub-PPI dataset will then include the proteins initially existing in the bicluster in addition to their interaction partners drawn from the considered PPI dataset as shown in Fig 2(b).

### Pruning Biclusters’ PPI Data

The biological approaches used to identify PPIs are very sensitive to experimental conditions and are thus susceptible to high error rates [4]. As a result, many methods were developed to filter PPI datasets in order to reduce the level of false positive and false negative interactions [16, 27, 28]. In our work, we use the PE method introduced by Zaki et al. in [16] to assess the reliability of protein interactions at the level of generated biclusters and prune the corresponding PPI subsets accordingly. Experiments show that PE-measure is efficient as it reduces the level of noise in protein interaction networks by looking for sub-graphs that are closest to maximal cliques, based on the weighted clustering coefficient measures, Fig 2(c).

### Detecting Protein Complexes

Successively, a protein-complex detection method is applied on the pruned biclusters’ PPIs, disjointedly on every bicluster. Subsequently, several sets of identified protein complexes are formed (*DC*_1_, *DC*_2_, …, *DC*_*k*_) as shown in Fig 2(d).

### Merging and Filtering the Detected Sets of Protein Complexes

Merging and filtering the resultant sets of complexes is crucial to the overall accuracy of our approach. However, developing an appropriate post-processing method is challenging because it is subject to various considerations. For instance, in its simplest form, it may consist of matching the detected entities against each other and combining the ones which have an overlap greater than a certain threshold. In contrast, keeping the common members of highly-overlapping entities may also be explored and it might lead to better outcomes. Another approach may think through the core-attachment interpretation of complexes [1] and consider that a repeated subgroup of interacting proteins in several detected groupings may be a potentially correct core, which forms different complexes when linked with various protein attachments. Nonetheless, in our paper, we keep this task for later research stages and we hereby limit the formation of the combined set of complexes to merging based on an overlap threshold and a condition by which members of one complex interact with a certain percentage of members of the other complex; in addition to filtering duplicates. This step finalizes the complex-detection process outlined by our framework, Fig 2(e).

## Experimental Study

### Datasets

DyCluster requires a gene expression dataset to model the dynamic aspect of protein interactions and a PPI dataset from which the interconnections among those proteins are extracted. Indeed, the higher the homogeneity of both sets, namely in terms of the species and the number of common genes that they cover, the better are the expected outcomes. We referred to Gene Expression Omnibus (GEO) repository [52] from which we selected the expression dataset of accession number GSE3431 [53], entitled “Logic of the yeast metabolic cycle”. It reports the expression levels of genes across twelve time intervals in three successive metabolic cycles. Our choice was primarily based on its wide coverage of yeast proteins and potentially, a high number of participants in various cellular processes. The yeast PPI dataset was downloaded from the Database of Interacting Proteins (DIP) [54] catalogue of experimentally-determined protein interactions. Finally, as reference set of yeast protein complexes with which we compared our results is the CYC2008 catalogue [55] containing 408 complexes.

### Experimental Settings

For the gene expression biclustering step, we used three algorithms: OPSM [50], CC [47] and ISA [51]. Here, we note that although efforts are spent in the direction of finding suitable ways to evaluate biclustering approaches [56], comparing their performances is still a challenging task. Added to that, in order to shed the light on the advantage of using gene expression data, we also examined the results of applying the framework using the one-way clustering method *k*-means [57]. The parameters settings of these algorithms are presented in Table 1. For the CC algorithm, as mentioned earlier, the Mean Square Residue (MSR) of a bicluster reflects the degree of coherence of the genes and the conditions contained in it. And, the lower the MSR, the higher is the coherence of the bicluster. Here, the upper limit of MSR is 0.5, by default. The threshold for multiple node deletion is used throughout the iterations of the algorithm to remove multiple nodes in the direction of lowering the MSR value of the generated biclusters. The number of output biclusters can also be specified for the CC method, here 10. While searching for large sub-matrices in which genes have the same linear ordering of the samples, the number of passed models at each iteration of the OPSM algorithm is set to 10, by default. The Iterative Signature Algorithm (ISA) identifies co-expressed genes across conditions based on thresholds for gene scores (*t*_*g*_) as well as condition scores (*t*_*c*_), both set to 0.5 by default. It also requires specifying the number of starting points for biclusters formation, here 100. The *k*-means clustering method takes as input parameters the number of clusters to be generated, set to 10, the number of iterations of the algorithm, set to 100, the number of replications, here 1, in addition to the distance measure used to calculate the level of expression similarity of genes, here Pearson’s correlation. We used the BicAT tool [58] to visualize and perform the biclustering of the gene expression dataset.

**Table 1:** Parameter settings of the applied biclustering algorithms.

Parameter Settings	
CC	upper limit of MSR: *δ* = 0.5
	threshold for multiple node deletion: *α* = 1.2
	number of output biclusters = 10
OPSM	number of passed models for each iteration: *l* = 10
ISA	threshold of genes: *t*_*g*_ = 0.5
	threshold of chips: *t*_*c*_ = 0.5
	number of starting points = 100
*k*-means	distance measure: Pearson’s correlation
	number of clusters = 10
	number of iterations = 100
	number of replications = 1

For the step consisting of pruning the PPI data at the biclusters levels, we adopted the PE method [16] with default parameters, specifically, with edges reliability score threshold equals to 0.1. In terms of protein-complex detection methods, we used ProRank [13], ProRank+ [15], ClusterONE [9] and CMC [7], MCODE [6] and CFinder [12]. ProRank, ProRank+, ClusterONE and CFinder were applied with default parameters.

Given a protein interaction network, CMC generates maximal cliques which may overlap. The highly-overlapping ones, i.e. with overlap greater than a specified threshold, are examined for possible merging if their degree of inter-connectivity exceeds a merging threshold. The overlap and merging thresholds were set to 0.75 and 0.5, respectively. For MCODE: the degree cutoff for a node to be scored was set to 2; the node score cutoff was set to 0.2, i.e. a node can be added to a cluster (complex) only if its score is no more than 20% less than the score of the seed node of the cluster; the *k*-core parameter, here set to 2, filters out clusters that do not contain a maximally inter-connected sub-cluster of at least degree *k*; and the maximum depth parameter which limits the distance from the seed node within which the algorithm can search for cluster members from seed was set to 3. Added to that, the generated sets of detected complexes were examined and refined as follows: if two complexes have a number of overlapping members greater than 75% of the size of the smaller complex; and if the members of the first complex interact with at least 50% of the members of the second complex, then they are merged.

### Evaluation Scores

The quality scores, used to evaluate our approach, included: (a) the number of complexes in the reference catalogue that are matched with at least one of the predicted complexes with an overlap score, OS ≥ 0.2; (b) the clustering-wise sensitivity (Sn) and (c) the clustering-wise positive predictive value (PPV) used to calculate the matching quality, mainly in terms of the correctly-matched protein members among the detected complexes; (d) the geometric accuracy (Acc) which is the geometric mean of Sn and PPV; and (e) the maximum matching ratio (MMR) which measures the maximal one-to-one mapping between predicted and reference complexes by dividing the total weight of the maximum matching with the number of reference complexes. Given *m* predicted complexes and *n* reference complexes, the corresponding formulas are shown in Table 2, where *t*_*ij*_ represents the number of proteins that are found in both predicted complex *m* and reference complex *n*.

**Table 2:** The formula of the quality scored used to evaluate our approach.

Evaluation Scores	Equations
Overlap score: between two protein complexes *A* and *B*	$OS\left(A,B\right)=\frac{{\left|A\cap B\right|}^{2}}{\left|A||B\right|}$
Clustering-wise sensitivity	$S_{n}=\frac{\sum_{i=1}^{n}max_{j=1}^{m}t_{ij}}{\sum_{i=1}^{n}n_{i}}$
Clustering-wise positive predictive value	$PPV=\frac{\sum_{j=1}^{m}max_{i=1}^{n}t_{ij}}{\sum_{j=1}^{m}\sum_{i=1}^{n}t_{ij}}$
Accuracy	$Acc=\sqrt{S_{n}\times PPV}$

## Results

According to the presented framework, the gene expression dataset, GSE3431, was processed by the three biclustering algorithms, OPSM, CC and ISA, and by the *k*-means clustering algorithm, one at a time. The PPIs corresponding to the proteins contained in each of the resulting biclusters were extracted from the specified yeast PPI dataset and were pruned using PE technique. The protein complex-detection methods, listed above, were applied on the generated biclusters. Finally, the detected sets of complexes were merged, filtered and matched against the CYC2008 reference catalogue.

In order to observe the advantage of our approach, Table 3 presents the results of detecting protein complexes in static PPI networks using various methods, i.e. without incorporating gene expression data. In contrast, Table 4 shows the outcomes corresponding results to our proposed approach. Results in both tables are in terms of the number of matched protein complexes and the number of detected complexes along with the corresponding evaluation scores.

**Table 3:** Experimental results of matching the detected sets of protein complexes by various detection methods against the CYC2008 reference catalogue.

Method	No. of matched complexes	No. of detected complexes	Acc	*S*_*n*_	MMR	PPV
ProRank	41	230	0.4715	0.3072	0.1032	0.7237
ProRank+	46	274	0.4788	0.3371	0.1161	0.6801
ClusterONE	76	365	0.6008	0.511	0.2349	0.7064
CMC	114	4292	0.6587	0.6517	0.347	0.6658
MCODE	62	168	0.55	0.4271	0.149	0.7082
CFinder	116	6381	0.6143	0.5641	0.3776	0.669

**Table 4:** Experimental results of matching the detected sets of protein complexes by our proposed framework against the CYC2008 reference catalogue in comparison to ProRank, ProRank+, ClusterONE, CMC, MCODE and CFinder.

Method	Biclustering Algorithm	No. of matched cmplxs	No. of detected cmplxs	*Acc*	*S*_*n*_	MMR	PPV
ProRank	OPSM	78	335	0.5911	0.4627	0.2103	0.755
	CC	63	252	0.5658	0.4296	0.1804	0.7451
	ISA	71	320	0.564	0.4332	0.195	0.7342
	*k*-means	71	331	0.556	0.4222	0.1896	0.7322
ProRank+	OPSM	81	397	0.5982	0.5116	0.225	0.6995
	CC	65	305	0.5668	0.4724	0.1947	0.6802
	ISA	78	392	0.5677	0.4719	0.2231	0.683
	*k*-means	78	424	0.5687	0.4782	0.2196	0.6764
ClusterONE	OPSM	89	929	0.6426	0.5758	0.2469	0.7172
	CC	78	578	0.6267	0.5465	0.2036	0.7186
	ISA	87	890	0.6015	0.5506	0.2499	0.6571
	*k*-means	83	862	0.6153	0.533	0.2334	0.7102
CMC	OPSM	100	1207	0.6159	0.5566	0.2903	0.6816
	CC	95	1145	0.5983	0.5264	0.2844	0.6801
	ISA	100	1843	0.6041	0.5518	0.3071	0.6614
	*k*-means	94	1126	0.6088	0.5542	0.2913	0.6689
MCODE	OPSM	71	475	0.5695	0.4602	0.1835	0.7049
	CC	60	285	0.545	0.4058	0.1581	0.7321
	ISA	63	315	0.5529	0.4232	0.171	0.7222
	*k*-means	74	448	0.5658	0.4583	0.1947	0.6986
CFinder	OPSM	94	2079	0.6187	0.525	0.2925	0.7291
	CC	98	1236	0.5977	0.559	0.3005	0.6391
	ISA	99	2119	0.5738	0.5393	0.3021	0.6104
	*k*-means	99	1352	0.5988	0.5455	0.3098	0.6574

As the experimental results show, the incorporation of gene expression data in the process of detecting protein complexes in dynamic PPI networks is indeed beneficial, in contrast with the outcomes of detecting complexes in static networks. On one hand, it could notably increase the number of matched complexes, as it is the case for ProRank, ProRank+ and ClusterONE. We note here that the total number of detected complexes increased. Nevertheless, the quality scores, which depend on this number and the number of matched complexes as well, were slightly better. The former underlines the effectiveness and the potential of our framework in terms of increasing the number of matches while also ameliorating the quality of the detected entities. Here, we recall the need to develop a more suitable approach for merging, filtering and refining the identified sets of complexes (the last step of the presented framework) which would potentially lead to enhanced evaluation scores. On the other hand, biclustering genes based on their expression patterns could significantly reduce the large number of complexes detected by some algorithms, such as CMC and CFinder, while not compromising the quality of the results.

**Fig 3:**
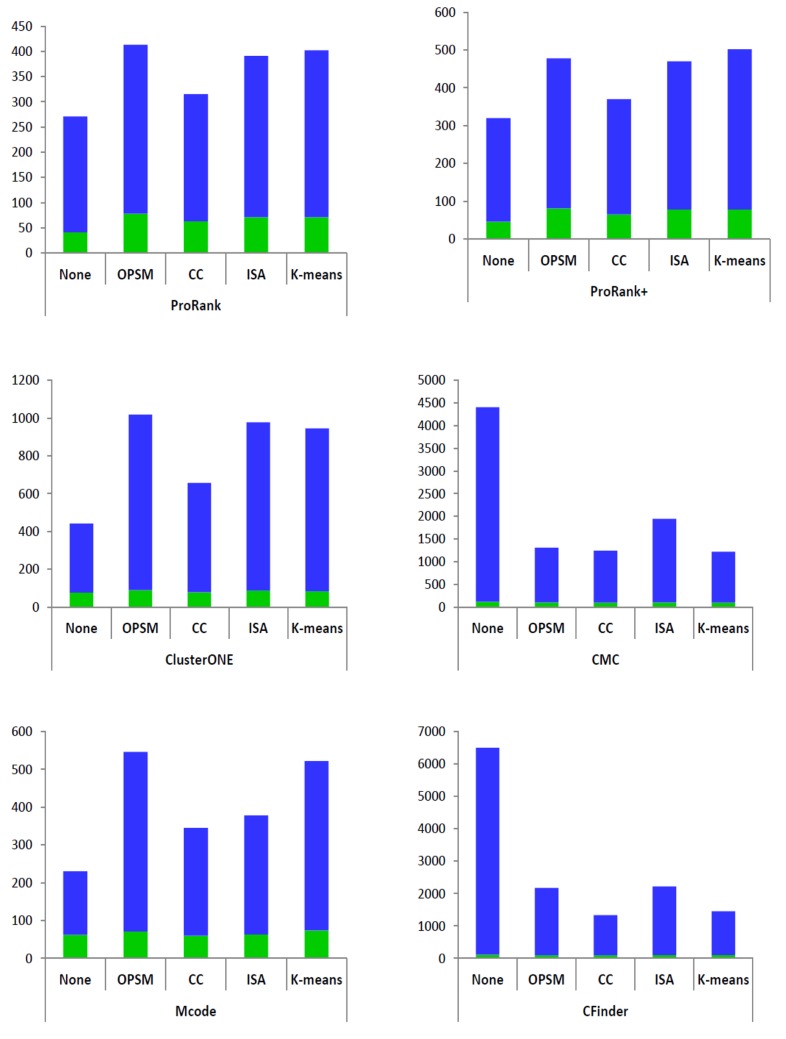
Statistical significance of scores differences between pairs of protein-complex detection methods without and with gene expression data based on the proposed framework. The displayed *p*-values are the ones less than or equal to 0.1 reflecting improvements in the scores, i.e. the matching qualities of the detected protein complexes.

We also examine the statistical significance of the improvements in the evaluation metrics (Acc, Sn, MMR and PPV). To do that, we perform a paired t-test to compare the results of just applying each complex-detection method on the PPI data, i.e. scores in each row of Table 3, with the scores corresponding to applying the framework with the same detection method and various biclustering algorithm (scores in Table 4). The samples are considered related since they are based on the same PPI data and reference set of protein complexes. Fig 3 shows the resulting p-values less than or equal to 0.1, they correspond to significant improvements given by the proposed framework. It is important to note that p-values tend to be lower when the difference in the sample means is higher. Although the mean differences among the considered scores are not high in this case, we can still note the reflected statistical significance of the improvements.

The conveyed study validates the enhancement of protein complex-detection approaches by integrating gene expression data, particularly through biclustering techniques. The framework models the dynamic aspect of PPI networks by grouping proteins according to the similarities of their expression patterns across subsets of conditions. Moreover, our method is not restricted by single threshold imposition on gene expression levels. As mentioned earlier, biclustering approaches are better than conventional clustering methods when it comes to expression data analysis [45, 46]. Nonetheless, the results attained by DyCluster using the *k*-means clustering algorithm accentuate the improvement which can be gained by incorporating gene expression information to model the dynamics of PPI interactions and to detect protein complexes in PPI networks accordingly.

### Testing DyCluster on Biological Data

In order to further test the effectiveness of the presented framework in identifying biologically related group of genes/proteins, we selected 140 pathway-focused genes implicated in programmed cell death in Rat Apoptosis and inflammation. The Rat Apoptosis RT^2^ Profiler PCR Array profiles the expression of 84 key genes (available at http://www.sabiosciences.com/rt_pcr_product/HTML/PARN-012Z.html) involved in programmed cell death. Apoptosis plays a critical role in normal biological processes requiring cell removal including differentiation, development, and homeostasis. Similarly, the Rat Inflammatory Cytokines and Receptors RT^2^ Profiler PCR Array profiles the expression of another 84 key genes (available at http://www.sabiosciences.com/rt_pcr_product/HTML/PARN-011Z.html) mediating the inflammatory response. Acute inflammation occurs in response to cell damage due to infection or injury. During this process, cellular and plasma derived factors encourage extravasation, the recruitment of circulating immune cells into the affected tissue. The two set of genes which are relevant to liver cancer are then combined and housekeeping genes and redundant genes are removed. Monitoring the expression of these genes helps to determine the mechanisms behind programmed cell death. The genes are then processed using String 9.1 [59] (Search Tool for the Retrieval of Interacting Genes/Proteins). String is a biological database and web resource of known and predicted protein-protein interactions. Genes with no records in String 9.1 were removed and therefore, 140 genes were considered. All proteins and their interactions were retrieved and the corresponding network was built. Once the PPI network (1,413 interactions and 140 proteins) was built, several enrichment features available in String 9.1 (features related to KEGG pathway, Reactome Pathway, Molecular function, Pfam domain, InterPro-Domains) were used to generate several sub-networks/groups which were then treated as protein complexes. The idea here is to see whether DyCluster is capable of detecting such groups of biologically-related proteins given only the PPI network information.

In this experimental work, the gene expression dataset, of accession number GSE17384, was downloaded from the GEO [52] repository. It is entitled: “Gene expression data from the LEC rat model with naturally occuring and oxidative stress induced liver tumorigenesis” [60]. It reports the variations of gene expression levels in a stepwise manner from the normal liver condition, to chronic induced liver tumor by time-series microarray analysis. In other words, the study involves a comparison between normal liver tissues and developed liver tumors at different time points. It could potentially reveal genes which participate in the progressive formation of the disease. The OPSM method [50] was used to bicluster the gene expression data since it showed a relatively good performance in our experimental study.

The PPI dataset was deduced from two sets of genes involved in apoptosis (RT^2^ Profiler PCR Array Rat Apoptosis, PARN-012A. The ProRank+ algorithm was employed to detect the corresponding protein entities/complexes. Then, we examined the generated results for potential matching with the reference sub-networks/groups generated using String. Table 5 shows the detected components by DyCluster framework, listed by types, along with their matching percentages. The experimental results thus confirm the potential of our approach in detecting and understanding protein entities of key roles in normal and abnormal cellular functions.

**Table 5:** The biological components detected by our framework, listed by types, along with their matching percentages.

	Detected Component	Matching Percentage
InterPro-Domains	Chemokine receptor family	100
	G protein-coupled receptor, rhodopsin-like	100
	GPCR, rhodopsin-like, 7TM	100
	BLC2 family	83.3
	BLC2-like	83.3
	Death effector domain	66.7
	Interleukin-6 receptor alpha, binding	50
	Death domain	100
	Apoptosis regulator, Bcl-2, BH2 motif, conserved site	75
	Chemokine interleukin-8-like domain	60
KEGG Pathway	Chemokine signaling pathway	40
	Cytokine-cytokine receptor interaction	32.8
	NOD-like receptor signaling pathway	31.3
	Apoptosis	34.4
	Autoimmune thyroid disease	71.4
	Huntington’s disease	66.7
	Systemic lupus erythematosus	40
	Asthma	50
	Intestinal immune network for IgA production	25
	Cell adhesion molecules	50
	Pathways in cancer	70
Molecular Function	Peptide receptor activity	58.3
	Receptor activity	52.2
	Growth factor activity	60
	C-C chemokine binding	66.7
	Tumor necrosis factor receptor superfamily binding	40
	Death effector domain binding	66.7
	Growth factor binding	50
	Nucleic acid binding transcription factor activity	75
	Chemokine activity	77.8
Pfam Domains	7 transmembrane receptor, rhodopsin family	100
	Apoptosis regulator proteins, Bcl-2 family	83.3
	Death effector domain	66.7
	Interleukin-6 receptor alpha chain, binding	50
	Small cytokines (intecrine/chemokine), interleukin-8 like	53.3
	Death domain	100
Reactome Pathway	Activation of DNA fragmentation factor	66.7
	Interleukin-1 family precursors are cleaved by caspase-1	100
	Downstream TCR signaling	100
	FasL/CD95L signaling	100
	Exocytosis of platelet alpha granule contents	100
	IRAK4 is activated by autophosphorylation	75
	Beta defensins	66.7
	TRAIL signaling	66.7
	Interleukin-1 processing	75
	FASL:FAS Receptor Trimer, FADD complex	100

## Discussion

**Fig 4:**
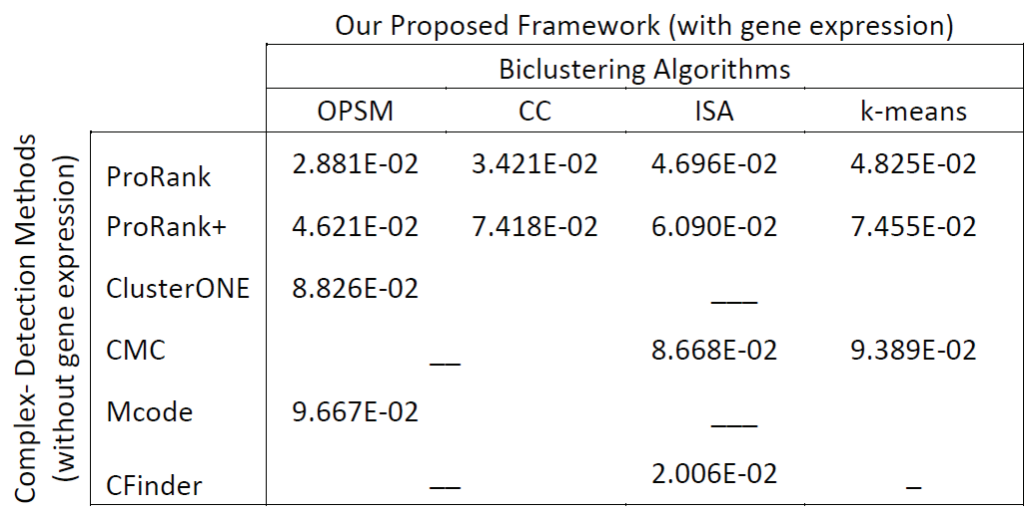
The number of matched (in green) and detected (in blue) complexes per detection method.

DyCluster was tested using several biclustering techniques and various protein complex detection methods. As the experimental results show, the incorporation of gene expression data in the process of detecting protein complexes in dynamic PPI networks is indeed beneficial, in contrast with the detection of complexes in static networks. Fig 4 shows the number of matched and detected complexes per detection method presented in Tables 3 and 4. It can be noticed that on one hand, our framework can notably increase the correctness and the quality of the results, as it is the case for ProRank, ProRank+ and ClusterONE where the numbers of matched complexes, Acc, Sn, PPV and MMR are higher. On the other hand, biclustering genes based on their expression patterns can significantly reduce the large number of complexes detected by some algorithms, such as CMC and CFinder, while not compromising the quality of the outcomes. The framework models the dynamic aspect of PPI networks by grouping proteins according to the similarities of their expression patterns across subsets of conditions. Moreover, it is not restricted by threshold imposition on gene expression levels. As mentioned earlier, biclustering approaches are better than conventional clustering methods when it comes to expression data analysis. Nonetheless, the results attained by DyCluster using the *k*-means clustering algorithm accentuate the improvement which can be gained by incorporating gene expression information to model the dynamics of PPI interactions and to detect protein complexes in PPI networks accordingly. Finally, the produced results based on the case study shown Table 5 are in favor of the DyCluster framework.

## Conclusion

DyCluster is a framework for the detection of protein complexes in dynamic protein interaction networks modeled by incorporating gene expression data, through biclustering techniques. It responds to the important shift from interpreting PPI data as a single static network to modeling and exploring the dynamic nature of these networks. That is done by incorporating gene expression data, interpreted using biclustering techniques, in the interaction networks and detecting complexes accordingly. The experimental results greatly favor our approach which allows the correct identification of more protein complexes. Moreover, in cases where this is not attained, the overall number of detected complexes is decreased and this leads to better evaluation scores. Hypothetically, the more biological information is added to PPI networks, the better the interaction dynamics are reflected. Therefore, and based on our results, further extensions consist of refining the modeling of PPI dynamics using additional biological data types.

## References

[1] Gavin A.C. et al Proteome survey reveals modularity of the yeast cell machinery. *Nature*, 440:631–636, 2006 10.1038/nature04532 16429126

[2] Fields S. and Song O. A novel genetic system to detect protein-protein interactions. *Nature*, 340:245–246, 1989 10.1038/340245a0 2547163

[3] Collins M.O. and Choudhary J.S. Mapping multiprotein complexes by affinity purification and mass spectrometry. *Curr. Opin. Biotechnol*., 19:324–330, 2008 10.1016/j.copbio.2008.06.002 18598764

[4] Adelmant G. and Marto J.A. Protein complexes: the forest and the trees. *Expert Rev. Proteomics*, 6(1):5–10, 2009 10.1586/14789450.6.1.5 19210120

[5] Dongen S. *Graph clustering by flow simulation*. University of Utrecht, 2000.

[6] Bader G.D. and Christopher W.H. An automated method for finding molecular complexes in large protein interaction networks. *BMC Bioinformatics*, 4:2, 2003 10.1186/1471-2105-4-2 12525261 PMC149346

[7] Guimei L., Wong L., and Chua H.N. Complex discovery from weighted ppi networks. *Bioinformatics*, 25(15):1891–1897, 2009 10.1093/bioinformatics/btp311 19435747

[8] Frey B.J. and Dueck D. Clustering by passing messages between data points. *Science*, 315:972–976, 2007 10.1126/science.1136800 17218491

[9] Nepusz T., Yu H., and Paccanaro A. Detecting overlapping protein complexes in protein-protein interaction networks. *Nature Methods*, 9:471–472, 2012 10.1038/nmeth.1938 22426491 PMC3543700

[10] Andrew D.K., Przulj N., and Jurisica I. Protein complex prediction via cost-based clustering. *Bioinformatics*, 20(17):3013–3020, 2004 10.1093/bioinformatics/bth351 15180928

[11] Przulj N., Wigle D.A., and Jurisica I. Functional topology in a network of protein interactions. *Bioinformatics*, 20(3):340–348, 2004 10.1093/bioinformatics/btg415 14960460

[12] Adamcsek B., Palla G., Farkas I.J., Derenyi I., and Vicsek T. CFinder: locating cliques and overlapping modules in biological networks. *Bioinformatics*, 22(8):1021–1023, 2006 10.1093/bioinformatics/btl039 16473872

[13] Zaki N.M., Berengueres J., and Efimov D. Detection of protein complexes using a protein ranking algorithm. *Proteins: Structure, Function and Bioinformatics*, 80(10):2459–2468, 2012 10.1002/prot.24130 22685080

[14] Zaki N.M. and Berengueres J. and Efimov D. ProRank: a method for detecting protein complexes. *Genetic and Evolutionary Computation Conference, GECCO’12, Philadelphia, PA, USA, July* 7–11, 2012, 209–216, 2012.

[15] Hanna E.M. and Zaki N.M. ProRank+: A method for detecting protein complexes in protein interaction networks. In *Bioinformatics 2014 - Proceedings of the Int. Conference on Bioinformatics Models, Methods and Algorithms, ESEO, Angers, Loire Valley, France*, 3-6 *March*, 2014, pages 239–244, 2014.

[16] Zaki N.M., Efimov D., and Berengueres J. Protein complex detection using interaction reliability assessment and weighted clustering coefficient. *BMC Bioinformatics*, 14:163, 2013 10.1186/1471-2105-14-163 23688127 PMC3680028

[17] Levy E.D. and Pereira-Leal J.B. Evolution and dynamics of protein interactions and networks. *Curr. Opin. Struct. Biol*., 18:349–357, 2008 10.1016/j.sbi.2008.03.003 18448325

[18] Przytycka T.M. Singh M. Slonim D.K. Toward the dynamic interactome: it’s about time. *Briefings in Bioinformatics*, 11:15–29, 2010 10.1093/bib/bbp057 20061351 PMC2810115

[19] Kim T.H. and Ren B. Genome-wide analysis of protein-DNA interactions. *Annu. Rev. Genomics Hum. Genet*., 7:81–102, 2006 10.1146/annurev.genom.7.080505.115634 16722805

[20] Johnson D.S., Mortazavi A., Myers R.M., and et al Genome-wide mapping of in vivo protein-DNA interactions. *Science*, 317:1497–1502, 2007 10.1126/science.1141319 17540862

[21] Loven J., Orlando D.A., Sigova A.A., and et al Revisiting global gene expression analysis. *Cell*, 151(3):476–482, 2012 10.1016/j.cell.2012.10.012 23101621 PMC3505597

[22] de Lichtenberg U., Jensen L.J., Brunak S., and et al Dynamic complex formation during the yeast cell cycle. *Science*, 307:724–727, 2005 10.1126/science.1105103 15692050

[23] Ashburner M., Ball C.A., Blake J.A., and et al Gene ontology: tool for the unification of biology. *Nat. Genet*., 25:25–29, 2000 10.1038/75556 10802651 PMC3037419

[24] Kim Y., Han S., Choi S., and et al Inference of dynamic networks using time-course data. *Briefings in Bioinformatics*, 15(2):212–228, 2013 10.1093/bib/bbt028 23698724

[25] Lee Y.H., Tan H.T., and Chung M.C. Subcellular fractionation methods and strategies for proteomics. *Proteomics*, 10(3935):3935–3956, 2010 10.1002/pmic.201000289 21080488

[26] Xu B., Lin H., and Yang Z. Ontology integration to identify protein complex in protein interaction networks. *Proteome Sci*., 9:S7, 2011 10.1186/1477-5956-9-S1-S7 22165991 PMC3289085

[27] Chua H. et al Using indirect protein-protein interactions for protein complex predication. *J. Bioinform. Comput. Biol*., 6:435–466, 2008 10.1142/S0219720008003497 18574858

[28] Hon N.C., Sung W.K., and Wong L. Exploiting indirect neighbours and topological weight to predict protein function from protein-protein interactions. *Bioinformatics*, 22(13):1623–1630, 2006 10.1093/bioinformatics/btl145 16632496

[29] Spirin V. and Mirny LA. Protein complexes and functional modules in molecular networks. *PNAS*, 100:12123–12128, 2003 10.1073/pnas.2032324100 14517352 PMC218723

[30] Zaki N.M. and Mora A. A comparative analysis of computational approaches and algorithms for protein subcomplex identification. *Scientific Reports*, 4:4262, 2014 10.1038/srep04262 24584908 PMC3939454

[31] Wang J., Peng X., Xiao Q., and et al An effective method for refining predicted protein complexes based on protein activity and the mechanism of protein complex formation. *BMC Systems Biology*, 7:28, 2013 10.1186/1752-0509-7-28 23537347 PMC3648373

[32] Wang J., Peng X., and Li M. Active protein interaction network and its application on protein complex detection. *In Proceedings of IEEE International Conference on Bioinformatics and Biomedicine (BIBM)*, pages 37–42, 2011.

[33] Li M., Chen W., Wang J., Wu F., and Pan Y. Identifying dynamic protein complexes based on gene expression profiles and PPI networks. *Biomed Research International*, 2014, 2014 10.1155/2014/375262 PMC4052612 24963481

[34] Friedman N., Linial M., Nachman I., and et al Using bayesian networks to analyze expression data. *J. Comp. Biol*., 7:601–620, 2007 10.1089/106652700750050961 11108481

[35] Remondini D., O’Connell B., Intrator N., and et al Targeting c-Myc-activated genes with a correlation method: detection of global changes in large gene expression network dynamics. *PNAS*, 102:6902–6906, 2005 10.1073/pnas.0502081102 15867157 PMC1100785

[36] Song L., Kolar M., and Xing E.P. KELLER: estimating time-varying interactions between genes. *Bioinformatics*, 25:i128–i136, 2009 10.1093/bioinformatics/btp192 19477978 PMC2687946

[37] Bansal M., Belcastro V., Ambesi-Impiombato A., and et al How to infer gene networks from expression profiles. *Mol. Syst. Biol*., 3(122), 2007 10.1038/msb4100120 17299415 PMC1828749

[38] Morris M.K., Saez-Rodriguez J., Sorger P.K., and et al Logic-based models for the analysis of cell signaling networks. *Biochemistry*, 49:3216–3224, 2010 10.1021/bi902202q 20225868 PMC2853906

[39] Lubovac Z., Gamalielsson J., Olsson B., and et al Combining functional and topological properties to identify core modules in protein interaction networks. *Proteins*, 64:948–959, 2006 10.1002/prot.21071 16794996

[40] Baldi P. and Hatfield G.W. DNA Microarrays and gene expression: From experiments to data analysis and modeling. Cambridge: Cambridge University Press, 2002.

[41] Watson J.D. DNA the secret of life. New York: Alfred A. Knopf, 2003.

[42] Asyali M.H., Colak D., Demirkaya O., and et al Gene expression profile classification: A Review. *Curr. Bioinformatics*, 1:55–73, 2006 10.2174/157489306775330615

[43] Jiang D., Tang C., and Zhang A. Cluster analysis for gene expression data: A Survey. *IEEE Trans. Knowl Data Eng*, 16(11):1370–1386, 2004 10.1109/TKDE.2004.68

[44] Hodgkin J. Seven types of pleiotropy. *International Journal of Developmental Biology*, 42(3):501–505, 1998 9654038

[45] Madeira S.C. and Oliveira A.L. Biclustering algorithms for biological data analysis: A Survey. *IEEE Trans Comput Biol Bioinf*, 1:24–25, 2004 10.1109/TCBB.2004.2 17048406

[46] Busygin S., Prokopyev O.A., and Pardalos P.M. Biclustering in data mining. *Comput OR*, 35(9):2964–2987, 2008 10.1016/j.cor.2007.01.005

[47] Cheng Y. and Church G.M. Biclustering of expression data. In *Proceedings of the 8th International Conference on Intelligent Systems for Molecular Biology, La JollaAAAI*, pages 93–103, 2000. 10977070

[48] Doruk Bozdağ, Ashwin S. Kumar, and Umit V. Catalyurek. Comparative analysis of biclustering algorithms. In *Proceedings of the First ACM International Conference on Bioinformatics and Computational Biology*, BCB’10, pages 265–274, New York, NY, USA, 2010. ACM.

[49] Mukhopadhyay A., Maulik U., and Bandyopadhyay S. A novel coherence measure for discovering scaling biclusters from gene expression data. *J Bioinformatics Comput Biol*, 7(5):853–868, 2009 10.1142/S0219720009004370 19785049

[50] Ben-Dor A., Chor B., Karp R.M., and et al Discovering local structure in gene expression data: the order-preserving submatrix problem. *J Comput Biol*, 10(3/4):373–384, 2003 10.1089/10665270360688075 12935334

[51] Bergmann S., Ihmels J., and Barkai N. Iterative signature algorithm for thebanalysis of large-scale gene expression data. *Phys Rev E Stat Nonlin Soft Matter Phys*, 67:03190201–03190218, 2003. 10.1103/PhysRevE.67.031902 12689096

[52] Barrett T., Wilhite S.E., Ledoux P., and et al NCBI GEO: archive for functional genomics datasets–update. *Nucleic Acids Res*., 41:D991–5, 2013 10.1093/nar/gks1193 23193258 PMC3531084

[53] Tu B.P., Kudlicki A., Rowicka M., and et al Logic of the yeast metabolic cycle: temporal compartmentalization of cellular processes. *Science*, 18(310):1152–8, 2005 10.1126/science.1120499 16254148

[54] Xenarios I., Salwinski L., Duan X.J., and et al DIP: The database of interacting proteins. a research tool for studying cellular networks of protein interactions. *Nucleic Acids Res*., 30:303–5, 2002 10.1093/nar/30.1.303 11752321 PMC99070

[55] Pu S., Wong J., Turner B., and et al Up-to-date catalogues of yeast protein complexes. *Nucleic Acids Res*., 37(3):825–31, 2009 10.1093/nar/gkn1005 19095691 PMC2647312

[56] Oghabian A., Kilpinen S., Hautaniemi S., and et al Biclustering methods: Biological relevance and application in gene expression analysis. *PLoS ONE*, 9(3):e90801, 2014 10.1371/journal.pone.0090801 24651574 PMC3961251

[57] Hartigan J. and Wong M. A K-Means clustering algorithm. *JR Stat Soc*, 28:100–108, 1979.

[58] Barkow S., Bleuler S., Prelic A., Zimmermann P., and Zitzler E. BicAT: a biclustering analysis toolbox. *Bioinformatics*, 21(10):1282–1283, 2006 10.1093/bioinformatics/btl099 16551664

[59] Jensen L.J. and et al String 8–a global view on proteins and their functional interactions in 630 organisms. *Nucleic Acids Res*, 37:D412–6, 2009 10.1093/nar/gkn760 18940858 PMC2686466

[60] Amin A., Hamza A., Bajbouj K., Ashraf S.A., and Daoud S. Saffron: A potential candidate for a novel anti-cancer drug against hepatocellular carcinoma *Hepatology*, 54(3): 857–867, 2011 10.1002/hep.24433 21607999

